# Development of morning–eveningness in adolescence: implications for brain development and psychopathology

**DOI:** 10.1111/jcpp.13718

**Published:** 2022-11-03

**Authors:** Rebecca Cooper, Maria A. Di Biase, Bei Bei, Nicholas B. Allen, Orli Schwartz, Sarah Whittle, Vanessa Cropley

**Affiliations:** ^1^ Melbourne Neuropsychiatry Centre The University of Melbourne and Melbourne Health Melbourne Victoria Australia; ^2^ Psychiatry Neuroimaging Laboratory, Department of Psychiatry, Brigham and Women's Hospital Harvard Medical School Boston Massachusetts USA; ^3^ Turner Institute for Brain and Mental Health, School of Psychological Sciences Monash University Melbourne Victoria Australia; ^4^ Department of Psychology University of Oregon Eugene Oregon USA; ^5^ Orygen Centre for Youth Mental Health Melbourne Victoria Australia

**Keywords:** Adolescence, psychopathology, sleep, morningness–eveningness, brain development

## Abstract

**Background:**

Morning–evening preference is defined as an individual's preference for a morning‐ or evening‐oriented rhythm. Across adolescence, a preference for eveningness becomes more predominant. Although eveningness is cross‐sectionally associated with internalizing and externalizing psychopathology, few studies have examined developmental changes in eveningness and its potential biological substrates. Here, we investigated the longitudinal relationships among the trajectory of eveningness preference, internalizing and externalizing psychopathology and white matter development, across adolescence.

**Methods:**

Two‐hundred and nine adolescents (49% male) were assessed longitudinally at four separate time points between 12 and 19 years of age. Morning–evening preference and internalizing and externalizing symptoms were assessed at each time point. Diffusion‐weighted images were acquired on a subset of participants at the final two time points to estimate changes in global mean fractional anisotropy (FA). Linear mixed models were performed to estimate the change in eveningness over time. A series of linear regression models assessed the influence of change in eveningness on psychopathology and white matter development at age 19.

**Results:**

Across the sample, a preference for eveningness became more predominant by 19 years of age. Greater individual‐level change towards eveningness significantly predicted greater severity in externalizing, but not internalizing, symptoms at 19 years of age. In contrast, change in psychopathology from 12 to 19 years of age was not associated with morning–eveningness at age 19. A change towards eveningness predicted an attenuated increase in FA between 17 and 19 years of age.

**Conclusions:**

This study suggests that developmental changes in morning–evening preference may predict both neurodevelopmental and psychological outcomes in adolescents.

## Abstract 2

Read the Commentary on this article at doi: 10.1111/jcpp.13747.

## Introduction

Adolescence is characterized by extensive changes in sleep behaviour and circadian rhythmicity (Carskadon, 2011). These changes are heavily influenced by a combination of physiological, neurodevelopmental and psychosocial factors, which lead to substantiative changes in the duration, timing and quality of sleep in adolescents (Owens et al., 2014). Among these changes is a shift in morning–evening preference (sometimes referred to as chronotype), defined as an individual's preference for a morning‐ or evening‐oriented rhythm (Adan et al., 2012). Individuals with a stronger preference for eveningness, for example, report ‘feeling optimal’ and most productive in the evenings (Adan et al., 2012), in addition to displaying objective delays in circadian rhythm (Adan et al., 2012; Kantermann et al., 2015). Previous cross‐sectional studies demonstrate that a preference for eveningness increases throughout early adolescence, peaking at approximately 17–19 years of age, before beginning to shift back towards more morningness in early adulthood (Randler et al., 2017). In addition, the trend towards eveningness appears maximal in early adolescence, coinciding with the onset of puberty (Randler et al., 2017). This suggests the presence of developmentally sensitive periods in changes in morning–evening preference, which may have implications for adolescent health and behaviour.

Crucially, adolescents with a stronger eveningness preference are considered at risk for a range of poor physiological, academic and mental health‐related outcomes, including an increased risk of experiencing symptoms of internalizing and externalizing psychopathology (Au & Reece, 2017; Merikanto et al., 2017; Norbury, 2021; Schlarb et al., 2014; Urrila et al., 2015). Nevertheless, while cross‐sectional relationships between eveningness and psychopathology are generally well characterized, considerably less work has examined prospective associations between chronotype and psychopathology during adolescence. Of such published studies, a stronger self‐reported preference for eveningness has been shown to bidirectionally relate to aspects of both internalizing and externalizing disorders, including depressive symptoms and substance use (Haraden et al., 2017, 2019; Hasler, Franzen, et al., 2017; Haynie et al., 2018; Nguyen‐Louie et al., 2018). However, given the transient nature of morning–evening preference across adolescence, it is difficult to justify the validity of selecting a single time point as representative of morning–evening preference across the entire adolescent period.

An alternative approach would be to assess the overall trajectory, or intra‐individual change in morning–evening reference, as this shifts across adolescence. Given the substantial variability of the timing and extent of this change among adolescents, individual differences in the *trajectory* towards morning‐ or eveningness may be an important factor for later adolescent outcomes. To our knowledge, only one study has investigated the impact of developmental *change* in morning–evening preference on psychiatric symptoms in adolescents (Soehner et al., 2019). This study identified prospective relationships between increases in eveningness across adolescence and exacerbation of a diverse range of psychopathology symptoms in a transdiagnostic sample of youth. Whether intra‐individual changes in eveningness influence later psychopathology in typically developing youth remains unknown.

Despite substantial evidence for a relationship between stronger eveningness preference and psychopathology, the neurobiological substrates of this relationship are unclear. In addition to changes in sleep‐related behaviour, adolescence is also characterized by dynamic changes in brain structure. This includes progressive increases in white matter, considered the product of gradual myelination of nerve fibres (Paus et al., 2008), which is thought to influence overall processing efficiency and subsequently adolescent behaviour (Fields, 2008). However, the highly plastic nature of white matter during development renders it vulnerable to a range of environmental and endogenous stimuli (Dow‐Edwards et al., 2019), including sleep‐related phenomena (Jan et al., 2010). In addition, evidence from animal models suggests that a range of sleep‐related behaviours during developmentally sensitive periods may directly influence brain maturation, including myelination (Bellesi et al., 2018).

Collectively, these studies point to neurodevelopment as a mechanism linking sleep and adolescent outcomes (Galván, 2020), including, potentially, relations between morning–evening preference and psychopathology. Indeed, a growing volume of literature provides evidence of relationships between morning–evening preference and measures of both brain structure and function, including white matter microstructure (Rosenberg et al., 2014), cortical thickness (Rosenberg et al., 2018; Takeuchi et al., 2015) and reward‐related brain activity (Hasler, Casement, et al., 2017). In addition, dysfunctional sleep behaviours commonly experienced by evening‐oriented individuals (Vollmer et al., 2017), such as later sleep timing, poorer sleep quality and greater sleep variability, are also associated with variations in brain structure (Jalbrzikowski et al., 2021; Mulder et al., 2019; Telzer et al., 2015). However, these studies are all cross‐sectional, and thus unable to make inferences about brain *development*. To date, there are no longitudinal studies of sleep‐related behaviour and brain structure in adolescence. Such studies are required to examine the purported role of sleep in neurodevelopment, and as a mechanism linking sleep, morning–evening preference and adolescent psychopathology.

To this end, the present study investigated prospective relationships between the trajectory of morning–evening preference, internalizing and externalizing psychopathology and white matter development, across adolescence. Morning–evening preference and white matter structure were examined given these measures show substantial development over adolescence, with both measures increasing nonlinearly across this period. In addition, attenuated white matter development has been associated with an increased risk for increased psychopathology in adolescents (Vanes et al., 2020). The aims of the study were threefold. Firstly, we aimed to characterize longitudinal changes in morning–evening preference across adolescence. Given previous cross‐sectional studies, it was hypothesized that morning–evening preference would follow a nonlinear trajectory, characterized by increasing eveningness across 12–17 years, followed by a gradual plateau and maximum at approximately 17–19 years of age. Secondly, we aimed to assess the prospective relationship between developmental *changes* in morning–evening preference and both psychopathology and white matter development. It was hypothesized that a shift towards greater eveningness would be associated with higher internalizing and externalizing symptoms at age 19, in addition to an attenuation of white matter development across the ages of 17–19 years of age. Thirdly, we aimed to examine whether white matter development may mechanistically link changes in morning–eveningness with later psychopathology. We hypothesized that attenuated white matter development would mediate the relationship between a shift to greater eveningness and psychopathology.

## Methods

### Participants

Participant data were collected from a longitudinal cohort study (the Orygen Adolescent Development Study [OADS]). The specifics of data collection are noted elsewhere (Yap et al., 2008). Briefly, the study sample was selected from a community sample of adolescents living in Melbourne, Australia. Initial screening of 2,453 participants enabled the selection of a subset of individuals (*N* = 415) evenly distributed across a range of temperaments, with the aim of examining elements of risk and resilience to psychopathology during adolescence. This subset was invited to participate in the longitudinal study, with 245 individuals consenting to participate who did not meet the following exclusion criteria: learning disability, obesity, growth failure, chronic medical or neurological illness, any history of significant head injury, evidence of current or past substance use or eating disorder. Assessments were completed across 2004–2011, when participants were aged approximately 12, 15, 17 and 19 years respectively (see Table 1). From this sample, 209 (102 males) individuals completed the reduced Morningness–Eveningness Questionnaire (rMEQ) 1–4 times and were included as the final analytical sample. Fifty (24%) individuals had rMEQ data at one time point; *N* = 62 (30%) had data for two time points; *N* = 76 (36%) at three time points and *N* = 21 (10%) at all four time points. The average amount of time points available across all participants was 2.3 (*SE* 0.96). At waves 3 and 4 (T3 and T4), all participants were invited to participate in a magnetic resonance imaging (MRI) session including diffusion‐weighted imaging (DWI). Of these, 136 individuals consented to a scan at T3 and 105 at T4. Handling of missing data is described further in Appendix S1; see also Figure S1 for participant flow and attrition.

**Table 1:** Demographic features of the sample

Variable	Mean	*SD*	Min	Max
Male, *N*	102 (49%)			
T1 Age	12.45	0.43	11.37	13.6
T2 Age	15.01	0.44	13.74	16.18
T3 Age	16.65	0.6	15.01	18.29
T4 Age	18.91	0.47	17.3	19.96
Ancestry				
Central European	92%			
Chinese	8%			
T2 Puberty (Tanner)	4.13	0.67	2	5
T1 rMEQ	14.69	1.58	11	20
T2 rMEQ	13.35	1.37	10	16
T3 rMEQ	13.42	1.69	7	17
T4 rMEQ	13.39	1.69	7	16
T1 BAI	8.25	8.21	0	43
T2 BAI	8.88	8.53	0	41
T3 BAI	9.12	8.78	0	47
T4 BAI	7.06	8.18	0	39
T1 CESD	11.49	9.46	0	55
T2 CESD	9.49	7.59	0	43
T3 CESD	10.81	8.86	0	48
T4 CESD	11.07	9.68	0	44
T1 CBCL	9.57	8.51	0	49
T2 CBCL	9.29	8.42	0	42
T3 CBCL	9.14	9.61	0	49
T4 CBCL	7.57	8.55	0	47
T3 Average Global FA	0.49	0.03	0.45	0.74
T4 Average Global FA	0.5	0.01	0.45	0.52

Pubertal status at T2 assessed by self‐report Tanner stage. BAI, Beck Anxiety Inventory; CBCL, Child Behaviour Checklist, parent‐report, externalizing items only; CESD, Centre for Epidemiological Studies – Depression Scale; MRI, magnetic resonance image; rMEQ, reduced Morningness–Eveningness Questionnaire; T1‐4: time points 1–4.

Informed consent was obtained from all participants and their caregivers in accordance with the guidelines of the Human Research Ethics Committee of the University of Melbourne, Australia.

### Procedures

#### Questionnaires

Internalizing and externalizing symptoms were assessed at all time points. These consisted of the self‐report Center for Epidemiological Studies Depression Scale (CESD; Radloff, 1977) and the self‐report Beck Anxiety Inventory (BAI; Beck & Steer, 1990) for internalizing symptoms, and the caregiver report of the Child Behavior Checklist (CBCL; Achenbach, 1991), externalizing items subscale, for externalizing symptoms. Self‐reported externalizing symptoms were assessed via the Youth Self Report at the final time point (T4). Morning–eveningness was assessed at all time points via the reduced Morningness–Eveningness Questionnaire (rMEQ), a 5‐item scale developed from the Horne & Östberg Morningness–Eveningness Questionnaire (Adan & Almirall, 1991). Pubertal status was reported by participants at the second time point (T2; *M* = 14.83 years) by the five Tanner stage model of secondary sexual characteristics (Marshall & Tanner, 1969). Participants were given schematic drawings of the five Tanner stages to assist in their reports. Females rated themselves on breast development and pubic hair growth, and males rated themselves on genitalia development. The mean score of the two report scores (females) or single report score (males) was used to indicate the level of pubertal development. In supplemental analyses, we covaried for recent substance use. An estimate of the past 30‐day frequency of substance use (combined cigarettes, alcohol and cannabis) was obtained from items extracted from the Youth Risk Behavior Survey (Centers for Disease Control and Prevention, 2001).

#### Diffusion‐weighted imaging acquisition

Diffusion‐weighted images (DWI) were acquired at T3 and T4 on a 3T Siemens TIM Trio scanner at the Royal Children's Hospital, Melbourne, Australia. Images were obtained using a spin‐echo EPI sequence in 60 directions (*b* = 2000 s/mm^2^), with 10 × T2‐weighted *b* = 0 reference images for reference (repetition time = 8,800 ms, echo time = 99 ms and field of view 256 mm, resulting in 64 slices of slice thickness 2.0 mm and voxel dimensions = 2.0 × 2.0 × 2.0 mm). A structural T1 scan was obtained in the same session as an anatomical reference (repetition time = 1,900 ms, echo time = 2.24 ms, flip angle = 9° and field of view = 230 mm, resulting in 176 T1‐weighted contiguous slices with voxel dimensions = 0.9 × 0.9 × 0.9 mm).

#### DWI pre processing

Diffusion‐weighted images were processed prior to analysis using the FMRIB Software Library (FSL; Jenkinson et al., 2012). Following axis alignment, images were skull stripped with FSL's *bet* tool (Smith, 2002). Correction of head motion and eddy currents was completed with FSL's *eddy* function (Andersson & Sotiropoulos, 2016). Fraction anisotropy (FA) volumes were computed for the remaining scans by fitting a single tensor to the DWI data, using the *bedpostx* tool within FMRIB's Diffusion Toolbox (Behrens et al., 2003). Resulting FA images were fed into tract‐based spatial statistics (TBSS; Smith et al., 2006) to nonlinearly register individual FA maps to the FMRIB58_FA standard target and to create a mean skeletonized FA image across all participants. Images with excessive motion (>2 standard deviations from mean) or poor reconstruction post‐TBSS were removed from further analysis (*N* = 13 scans; *N* = 11 at T3, *N* = 2 at T4), which resulted in 125 usable scans at T3, and 103 scans at T4. *N* = 94 individuals (42 males) had usable scans for both time points. Post‐TBSS, the global mean skeletonized FA for each participant was extracted and used for statistical analyses.

#### White matter development

Given the lack of literature on the effects of sleep‐related behaviours on longitudinal changes in white matter, we did not have any a priori regions of interest to investigate in our analyses. As such, we investigated white matter development at the global level. This was estimated by calculating the annualized percentage change (APC) in global mean FA between the two time points, as follows:

$$\mathrm{APC}:\frac{\text{Mean}\;\mathrm{FA}\left(T4\right)-\text{Mean}\;\mathrm{FA}\left(T3\right)}{\text{Mean}\;\mathrm{FA}\left(T3\right)}*\frac{1}{\text{Time interval}}*100\%$$

where time interval represents the number of days between scans at ages 17 and 19 (T3 and T4). Positive APC scores indicate increases in FA, while negative scores indicate decreases in FA. In secondary analyses, APC was also assessed at the voxel level to ascertain region‐specific effects of changes in morning–evening preference on white matter development. In supplemental analyses, we also assessed global APC in mean diffusivity (MD), calculating the APC as above.

### Statistical analysis

#### Estimating changes in morning–evening preference

Longitudinal age‐related change in morning–evening preference was estimated using linear mixed models (LMMs), implemented using the Linear and Nonlinear Mixed Effects Models (*nlme*) package in R version 4.0.2 (R Core Team, 2020). LMMs provide an advantage over traditional repeated‐measures analyses as they make use of all available data across time points, even in the presence of missing data. The LMM tested for both linear and nonlinear (quadratic and cubic) fixed effects of age, with sex included as a covariate. The best‐fitting model was selected based on the Bayesian information criterion and visual inspection of regression plots; an analysis of variance (ANOVA) was utilized to compare model fits. Interactions between sex and age were not significant and were omitted from the final model. In LMMs, random effects represent individual‐level changes in the outcome measure, which can vary in their initial level (i.e. intercept) and rate of change (i.e. slope). In our model, random effects of intercept and (linear) slope were specified, as the specification of nonlinear random effects led to singularity in the model. From the final models, random slope terms were extracted, representing individual‐level changes in morning–evening preference over time. Additional LMMs of age‐related change in psychopathology variables were also modelled and random slopes extracted (see below).

#### Effect of change in morning–evening preference on psychopathology at 19 years

**Figure 1:**
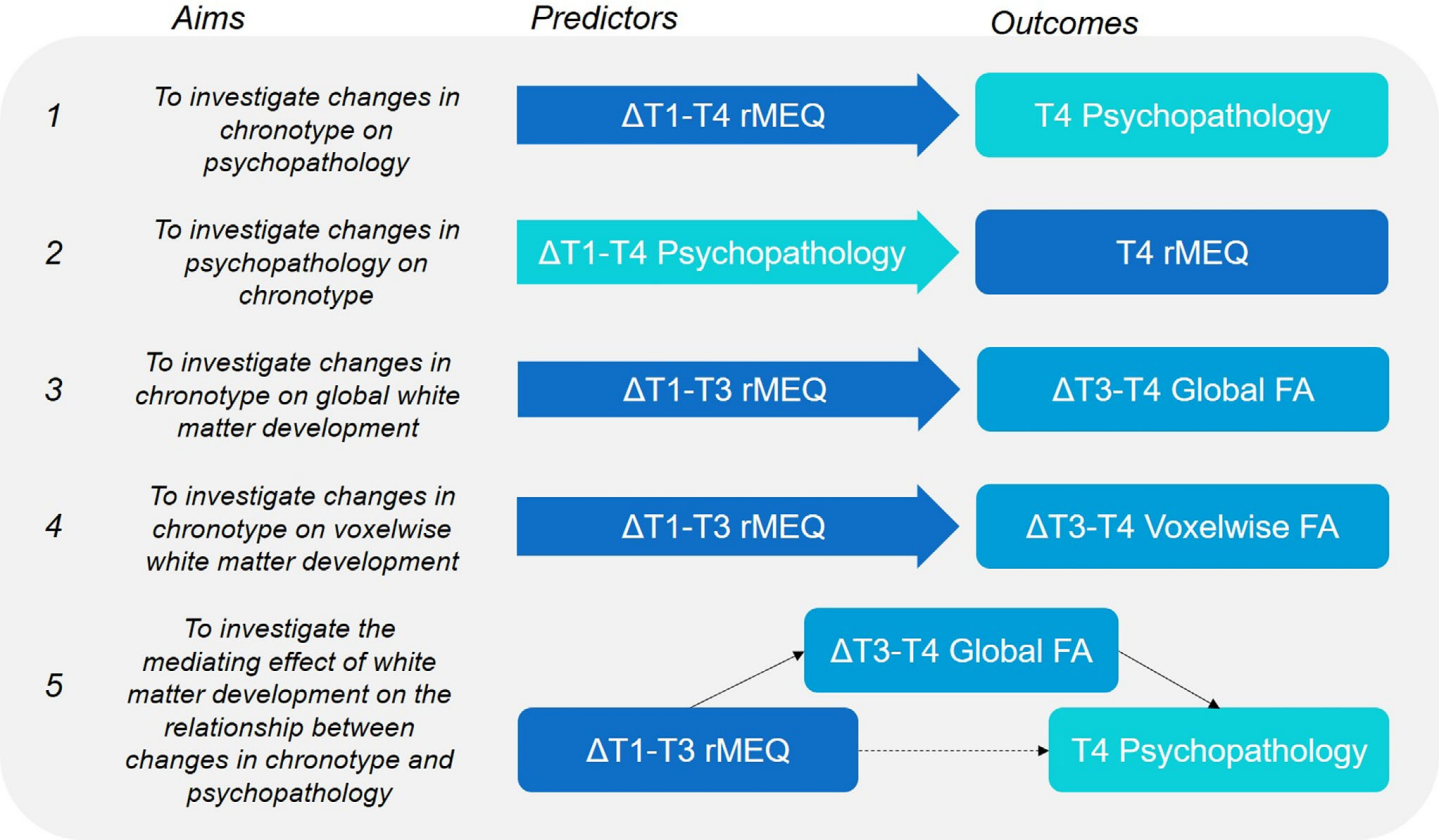
Diagrammatic summary of models. Main analyses are numbered (1–5) and the aims of each model are described in brief. Arrows represent model predictors, which were estimated by random slopes, as extracted from linear mixed models (LMMs). Changes in global and voxel‐wise FA were estimated by annualized percentage change (APC). T1–T4, time point 1–4; FA, fractional anisotropy; rMEQ, reduced Morningness–Eveningness Questionnaire [Color figure can be viewed at wileyonlinelibrary.com]

A summary of all models described hereafter is visualized in Figure 1. To examine whether individual changes in eveningness predicted outcomes relating to psychopathology (Figure 1, Model 1), a series of regression models were performed with the random slope term from the morning–eveningness LMM (above) as a predictor, and either of internalizing (BAI or CESD) or externalizing (CBCL) symptom scores at age 19 as outcome measures. To assess the reverse association (Figure 1, Model 2), the random slope term for each psychopathology variable (BAI, CESD and CBCL) served as predictors in regressions with morning–eveningness at age 19 as the outcome; separate models were run for each psychopathology variable and direction (6 models in total). Multiple comparisons were corrected with Benjamini and Hochberg's false discovery rate correction (Thissen et al., 2002; α ≤ 0.05). Each model adjusted for baseline (T1) psychopathology and sex, while the random slope estimation implicitly accounted for baseline rMEQ values. Likewise, for the reverse models, each adjusted for baseline rMEQ, and the psychopathology random slopes implicitly accounted for each baseline psychopathology score. The interaction between age and sex was not significant in each model and thus omitted. All regressions were implemented using Huber regressions via the *Modern Applied Statistics in S* (MASS) and *repmod* implemented in R version 4.0.2 (R Core Team, 2020). Huber regressions were utilized due to the presence of outliers in several predictors and outcome variables; Huber regressions are considered robust to outliers. Missing responses were removed pairwise in all analyses to maximize the available data.

#### Effect of change in morning–eveningness on white matter development

To examine the effect of changes in eveningness on white matter development (Figure 1, Model 3), a Huber regression model was run in a subset of participants with available DTI scans (*N* = 94), with change in eveningness between 12 and 17 years as predictor and APC in global FA across ages 17–19 as the outcome. Further analysis was run with voxel‐wise FA as the outcome measure to localize potential effects in white matter (Figure 1, Model 4). For each individual, APC was computed using the individual's FA volumes at ages 17 and 19 (T3 and T4), which resulted in a map that represented APC at every voxel traversing the white matter skeleton. On a voxel‐by‐voxel basis, a general linear model was performed to test the main effect of rMEQ trajectory on APC while controlling for sex. This was achieved with the *Randomise* tool in FSL, which conducts permutation‐based, nonparametric tests of variables of interest on MRI images (Winkler et al., 2014). Correction for multiple comparisons for the set of all skeleton voxels was performed with threshold‐free cluster enhancement (TFCE; Smith & Nichols, 2009). Additional supplemental analyses, including testing for directionality of effects and changes in average MD, are further detailed in the Supporting Information.

#### Mediation analysis

In the case of significant associations between change in morning–evening preference and both change in FA and psychopathology, mediation models were run to determine whether change in FA across age 17–19 (T3–T4) mediated the relationship between rMEQ trajectory and psychopathology at age 19 (T4; see Figure 1, Model 5). This analysis was conducted on a subset of participants with data on all predictors in this model after pairwise deletion (*N* observations = 75). The mediation model was performed using the PROCESS function for R (Hayes, 2017). The predictor was the variable representing individual changes in morning–evening preference between 12 and 17 years of age (T1–T3), the outcome was psychopathology score at age 19 (T4) and the mediator was the APC in global FA between ages 17 and 19 (T3–T4). Sex was used as a covariate. A bootstrapping method (*N* = 10,000 bootstrapped resamples) was used to test the significance of the mediation effect within a 95% confidence interval. Mediation effects were considered significant if the confidence intervals did not contain zero.

## Results

### Demographic information

A summary of the sample is provided in Table 1. Sensitivity analyses compared individuals with missing data on key outcome variables, and are outlined in Appendix S2 and Tables S1–S3. A flow chart of participants and sample attrition is provided in Figure S1.

### Morning–evening preference develops nonlinearly across adolescence

**Figure 2:**
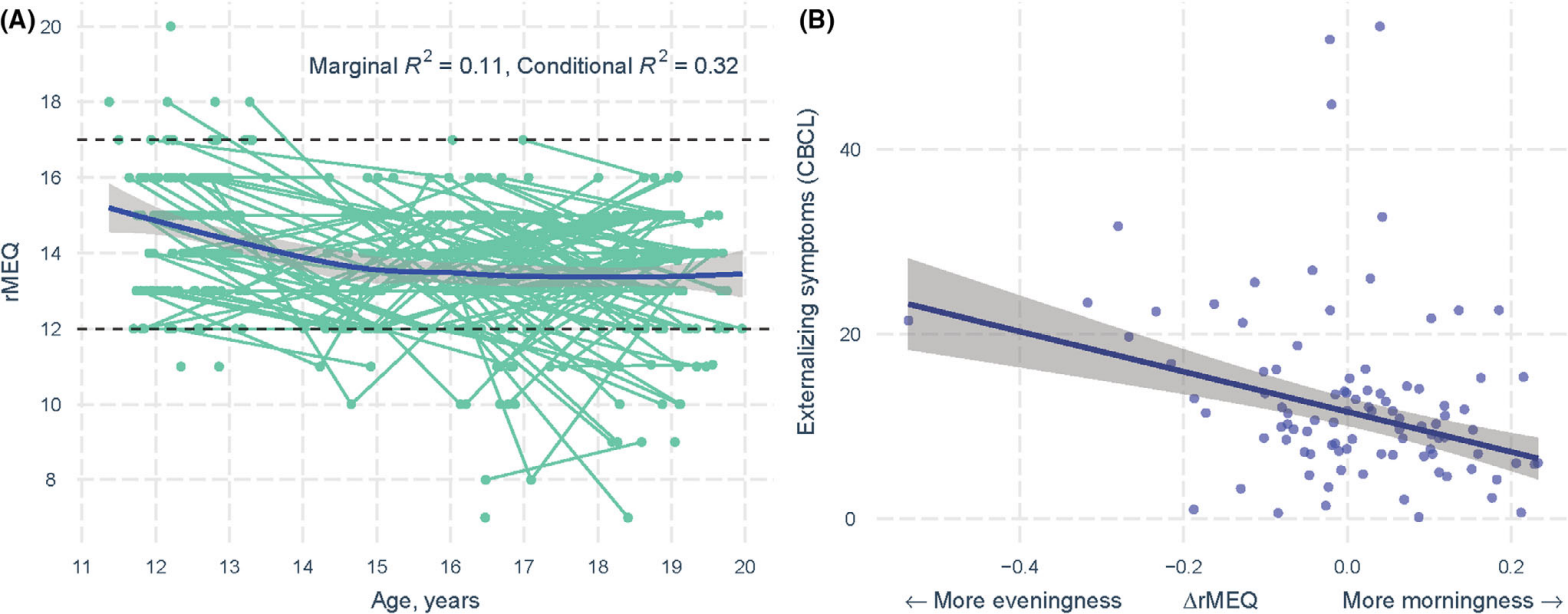
Longitudinal change in morning–evening preference across adolescence and relationship to externalizing symptoms. (a) Graph presents the change in morning–evening preference with age, including mean change (blue line) and individual‐specific change (green lines). Gray shading denotes 95% confidence intervals. Morning–evening preference measured with reduced Morningness‐Eveningness Questionnaire (rMEQ), where scores below 12 correspond to a strong evening preference, scores >17 correspond to a strong morning preference and intermediate scores between 12 and 17 correspond to neither strong morning nor evening preference. (b) The graph displays the relationship between change in morning–evening preference (ΔrMEQ) over early to mid‐adolescence, and parent‐reported externalizing scores at age 19. Gray shading denotes the 95% confidence interval and blue data points reflect individuals (*n* = 114). A positive change in rMEQ reflects a move towards more morningness across early to mid‐adolescence, whereas a negative change in rMEQ reflects a move towards more eveningness [Color figure can be viewed at wileyonlinelibrary.com]

Reduced Morningness–Eveningness Questionnaire score decreased significantly over adolescence, indicating increasing eveningness (Figure 2a, Table 2). The linear and quadratic effects of age on rMEQ were significant, and the quadratic model fitted the data significantly better than the linear model (Table S4). The cubic effect of age was not significant. Thus, the quadratic model was deemed the best‐fitting model for the data. This model indicated that, at a group level, eveningness increased with age following a positive quadratic slope (fixed effect of age^2^, *β* = 0.04 (*SE* 0.01), *t* = 3.00, *p* = .003), with a steeper decline seen in early adolescence (approximately 12–15 years) followed by a gradual plateau thereafter. Extraction of the fixed effect of age^2^, representing the group mean change morning–evening preference, yielded an estimated turning point for rMEQ score of 13.31 (95% CI 13.11–13.51), corresponding to an age of 17.76. This indicates that, at group level, the maximal preference for eveningness occurred at 17.76 years, before becoming more morning‐oriented thereafter. Including pubertal status in this model did not substantially alter the results (see Table S5). Regardless of this group‐level change, there were marked *individual* differences in morning–evening preference over time (Figure 2a). At each time point, the majority (88%–95%) of individuals were categorized as intermediate type (see Table S6). Fewer individuals were classified as morning type (0%–3%) and evening type (1%–12%) at each wave.

**Table 2:** Linear mixed‐effects model of the effect of age on morning–evening preference across adolescence

Predictors	Estimates	*SE*	Standardized *B*	*t*	*p*
Intercept	26.80	3.23	−0.02	8.30	**<.001**
Age	−1.49	0.42	−2.18	−3.51	.**001**
Age^2^	0.04	0.01	1.87	3.00	.**003**
Sex (Male)	0.06	0.18	0.04	0.35	.729
Marginal *R* ^2^/Conditional *R* ^2^ .114/.328					

Fixed effect of age, and squared effect of age and sex specified. Random effect of intercept and (linear) slope specified. *N* observations = 464; *N* groups = 209.

### Changes in morning–eveningness predict later psychopathology, but not the reverse

Random slope terms (representing individual‐level changes in morning–evening preference) were extracted from the above model and used as predictors of psychopathology symptoms at age 19. Individual‐level changes in morning–eveningness were found to predict externalizing symptoms at age 19 (Figure 2b, Table S7; *β* = −17.83 (*SE* 5.13), *t* = −3.48, *p* = .001), whereby increasing eveningness across adolescence predicted higher externalizing symptoms at age 19. In contrast, individual changes in morning–evening preference did not predict internalizing symptoms at age 19 (Table S8, BAI, *β* = −1.64 (*SE* 3.40), *t* = −0.48, *p* = .632; Table S9, CESD, *β* = −2.60 (*SE* 5.22), *t* = −0.50, *p* = .619). The inclusion of pubertal status at T2 as an added covariate did not alter the results (refer to Tables S10–S12). In reverse, individual‐level changes in psychopathology across ages 12–19 were not associated with eveningness preference at age 19 (all *p* > .05; Tables S13–S15). The inclusion of recent substance use (cigarettes, alcohol and cannabis) in the model predicting externalizing symptoms attenuated the effect sizes somewhat, but the relationships remained significant (Table S16). Further, individual‐level changes in morning–evening preference were also found to predict *child‐*reported externalizing symptoms at age 19 (Table S17). Supplementary analyses were also performed on the externalizing CBCL subscales (Delinquency and Aggressive Behavior) to determine whether increasing eveningness predicted specific externalizing symptoms or was generalized across the externalizing symptom dimension. Individual‐level change in morning–evening preference significantly predicted increases in both aggressive behaviour and delinquency (Tables S18 and S19).

### Individual change in morning–eveningness predict diffuse, nonspecific changes in global FA development

**Figure 3:**
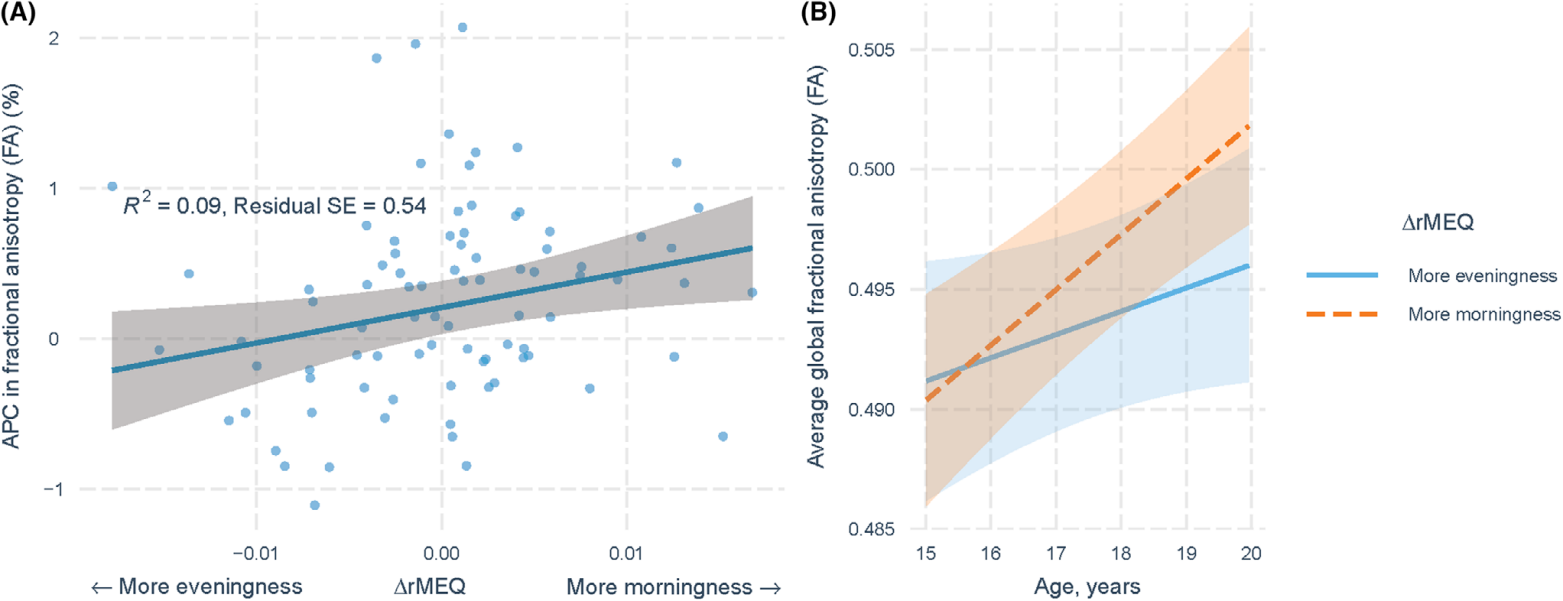
The relationship between longitudinal change in morning–evening preference and white matter development. (a) The graph displays the relationship between individual‐level change in morning–evening preference (ΔrMEQ) from age 12 to 17 on changes in fractional anisotropy (ΔFA) from age 17 to 19 years. The mean (blue line), 95% confidence intervals (grey shading) and individual data points (blue scatter) are shown. Positive changes in rMEQ (indicating an increase in morningness) were significantly associated with greater increases in FA over time. (b) To ease interpretation, change in rMEQ was converted to a categorical variable, where individuals were grouped according to whether they increased, overall, in morning or evening preference. These results highlight that increasing morningness predicts greater longitudinal increases in FA. Shading (orange and blue, respectively) denotes 95% confidence intervals [Color figure can be viewed at wileyonlinelibrary.com]

The annualized percentage change (APC) in FA across the ages of 17–19 years was 0.26% (*SD* 1.77). Change in morning–eveningness across ages 12–17 predicted change in FA from 17 to 19 (Figure 3a,b, Table S20; *β* = 22.87 (*SE* 9.67), *t* = 2.36, *p* = .020). A more positive random slope term, or a greater change towards morningness over time, was associated with a greater increase in FA. Conversely, a greater change towards eveningness was associated with a smaller increase in FA. The voxel‐wise analysis did not reveal any clusters significantly associated with rMEQ trajectory. Visual inspection of the FA skeleton revealed a diffuse, weak association between change in morning–eveningness and FA development across the entire skeleton. In supplemental analyses, change in morning–eveningness did not predict global MD values across ages 17–19 (see Table S21). Additionally, no significant relationship was observed for the reverse relationship, i.e. changes in APC in FA across ages 17–19 predicting morning–evening preference at age 19 (see Table S22).

### Global FA development did not mediate relationships between morning–evening preference and psychopathology

Mediation models were performed to examine whether white matter development mediated the relationship between individual changes in morning–evening preference and psychopathology at age 19. For the model predicting externalizing symptoms, both path a (rMEQ trajectory on the mediator, APC in FA) and path *b* (APC in FA on externalizing symptoms) were significant (*path a*, standardized *B* = 0.29, *t* = 2.56, *p* = .012; *path b*, standardized *B* = 0.28, *t* = 2.44, *p* = .017). The direct effect of change in morning–eveningness on externalizing symptoms (path c’) was also significant (standardized *B* = −0.26, *t* = −2.23, *p* = .029), and the standardized indirect effect (a*b) was 0.29*0.28 = 0.081. The bootstrapped standardized indirect effect was 0.082 (*SE* 0.06, 95% confidence interval −0.03 to 0.19). As the bootstrap confidence interval includes zero, the indirect effect is not significant; i.e. white matter development did not mediate the relationship between individual‐level changes in eveningness and externalizing symptoms at age 19. Supplemental analysis of whether white matter development mediated relationships between morning–eveningness trajectory and internalizing symptoms produced nonsignificant results (see Supporting Information).

## Discussion

In this longitudinal study, morning–evening preference increased in a nonlinear manner across the ages of 12 and 19 years, with a peak in eveningness occurring just prior to 18 years of age. Individual‐level increases in eveningness were also found to predict externalizing, but not internalizing symptoms, at age 19. Although increasing eveningness was associated with an attenuated trajectory of white matter development between 17 and 19 years of age, white matter development did not mediate the relationship between increasing eveningness and externalizing psychopathology. These results provide evidence of a relationship between developmental changes in eveningness and both brain development trajectories and later psychopathology during adolescence.

Consistent with cross‐sectional findings (Randler, 2011; Randler et al., 2017), our longitudinal study confirms that, at a group level, eveningness becomes more predominant across early adolescence, plateauing at a maximum level of eveningness at approximately 17–18 years of age. This nonlinear group trajectory occurred in the context of substantial intraindividual variability in morning–evening preference, with some individuals becoming more morning oriented, and others more evening oriented, over time. Our results complement recent work demonstrating that sleep timing, which correlates strongly with morning–evening preference (Vollmer et al., 2017), also evinces a nonlinear delay across adolescence (Karan et al., 2021). The previous study showed that sleep timing delayed between 15 and 19 years of age, before becoming earlier after the age of 19. Collectively, these findings suggest that population‐level morning–evening preferences might mirror developmental changes in sleep timing, with both measures evincing nonlinear changes prior to 19 years of age. Further studies should measure co‐occurring trajectories of morning–evening preference and objective sleep timing to ascertain the temporal relationship between these sleep‐related behaviours across development.

Individual‐level changes towards greater eveningness were found to predict increases in later parent‐ and child‐reported externalizing symptoms, including aggressive and delinquent behaviour. These findings are consistent with current literature (reviewed in Schlarb et al., 2014). These externalizing behaviours may relate to eveningness preference via a number of genetic, neurobiological and psychosocial pathways (Schlarb et al., 2014). Evening‐oriented adolescents are susceptible to circadian misalignment due to a mismatch between an endogenous circadian drive for later sleep timing and external societal pressures to rise early for school (Owens & Weiss, 2017). Substantial evidence links circadian misalignment to poorer mental health outcomes in adolescents, including a range of internalizing‐ and externalizing‐related outcomes (Nguyen et al., 2019). This mismatch contributes to adolescents sleeping later and longer on weekends to compensate for restriction throughout the week, resulting in increasing differences in weekday and weekend sleep, termed ‘social jetlag’ (Wittmann et al., 2006), itself an independent predictor of poor mental outcomes (Haynie et al., 2018; Mathew et al., 2019; Randler & Vollmer, 2013). In addition, emerging evidence suggests that circadian preferences, externalizing behaviours and sleep quality are influenced by common pleiotropic mechanisms (Barclay et al., 2011; Jones et al., 2019; Winiger et al., 2021). Further investigation of the mechanisms underlying these relationships is recommended in future work.

In contrast to our hypotheses, and the previous study by Soehner et al. (2019), increases in eveningness did not predict increases in internalizing symptoms at age 19. Our findings also contrast previous longitudinal studies demonstrating prospective, reciprocal relationships between evening preference and internalizing symptoms (Haraden et al., 2017). Nevertheless, our findings cohere with a similar‐sized study in adolescents reporting relationships between eveningness and externalizing, but not internalizing psychopathology (Merikanto et al., 2017). These discrepancies could be explained by differences in the age of psychopathology assessment; for example, the study by Haraden et al. (2017) examined individuals with a mean age of 12.08 years followed over 2.5 years, while our study, as well as that of Merikanto et al (who also reported null effects; Merikanto et al., 2017), examined psychopathology slightly later in adolescence. These findings, together with other work using this same dataset (unpublished), suggest that morning–evening preferences may preferentially influence internalizing symptoms in early, but not later, adolescence. We also posit that intraindividual *changes* in eveningness might predict only externalizing symptoms, while an individual's overall predisposition for eveningness might be a more relevant indicator for internalizing psychopathology. Future studies should seek to test this hypothesis, and also determine whether prospective relationships between eveningness preference and psychopathology are specific to discrete developmental periods across adolescence.

In contrast with previous work (Carskadon et al., 1993; Jankowski et al., 2019), we observed no relationship between morning–evening preference and puberty in our study. This may have been due to the limited amount of pubertal data available in this dataset. However, others have reported that age is a stronger predictor of morning–evening preference than pubertal status, although both are significant predictors of sleep timing (Randler et al., 2009). Further investigation is required to disentangle the effects of age and pubertal development on the development of morning–evening preference across adolescence, and the impact of pubertal status on the association between changes in morning–evening preference and later adolescent outcomes.

White matter structure increases nonlinearly across adolescence, paralleling improvements in information processing efficiency and cognitive capacity (Asato et al., 2010). Our findings indicate that changes in chronotype might influence this process. Compared to individuals moving towards a more morning‐oriented chronotype, individuals moving towards greater eveningness evinced an attenuated trajectory of white matter development over time. These findings complement a previous study in young adults, whereby evening‐type individuals displayed lower fractional anisotropy in frontal and cingulate white matter compared to morning‐oriented individuals (Telzer et al., 2015). Our findings indicate that these differences in white matter structure might emerge during the adolescent period, during which time the greatest shift to eveningness occurs (Randler et al., 2017). Taken together, this research implicates the role of chronotype in influencing white matter development through adolescence, potentially with implications into young adulthood.

Morning–evening preferences might relate to white matter development via several mechanisms. Substantial evidence indicates poorer behavioural and neurobiological outcomes related to eveningness occur via alterations in sleep‐related behaviour, given evening‐oriented individuals experience significantly shorter, more irregular and poorer‐quality sleep (Schlarb et al., 2014; Vollmer et al., 2017). In turn, poorer sleep health has been associated with alterations in brain structure in children and adolescents, such as lower fractional anisotropy (Mulder et al., 2019; Telzer et al., 2015), lower white matter volume (Kocevska et al., 2017) and cortical thickness (Jalbrzikowski et al., 2021). Growing evidence supports the role of sleep in developmentally sensitive periods of development, particularly for sensory‐ and motor‐related circuits (Frank et al., 2013). Sleep also plays a key role in supporting brain plasticity, which is upregulated during periods of brain development (Frank et al., 2013). Alternatively, eveningness preference could be related to white matter microstructure via circadian desynchrony and misalignment. As mentioned, evening‐type adolescents often experience circadian misalignment due to a mismatch between endogenous and external demands on sleep timing (Owens & Weiss, 2017). Findings from animal models indicate that deficits in brain structure occur in individuals exposed to chronic circadian dysrhythmicity, which occurs when external day/night cues are out of synchrony with an individual's endogenous circadian rhythm (Bedrosian & Nelson, 2017). Evening‐type adolescents more commonly experience this phenomenon due to early morning school starts which conflict with their delayed endogenous cycle (Owens et al., 2014). Many neurobiological systems are regulated by the endogenous circadian system, including the signalling and transcription of proteins involved in brain structure and plasticity. Desynchrony of the endogenous circadian rhythm could thus alter the rhythmic expression of these proteins, leading to downstream deficits in brain structural development (Bedrosian & Nelson, 2017). However, it is difficult to disentangle the direct effects of changes in sleep and chronotype on brain development, given their substantial overlap (Frank et al., 2013). Future work should seek to differentiate the specific effects of changes in sleep and chronotype on brain development in adolescence.

Consistent with previous findings in children and adolescents (Mulder et al., 2019; Rosenberg et al., 2014), morning–evening preferences were associated with white matter structure at a global level, rather than displaying spatial specificity. These findings are consistent with functional neuroimaging studies showing that morning–evening preference influences a range of behaviours associated with multiple brain regions, implicated in a range of diverse functions. For example, intraindividual variability in morning–evening preference has been shown to influence performance on tasks relating to memory consolidation, emotional and behavioural regulation, concentration, alertness and attention (Adan et al., 2012), which are regulated by spatially diverse neural circuits. Taken together, these findings support an association between individual morning–evening preference and systemic changes in white matter development, in contrast to displaying discrete regions of specificity. Nevertheless, given the discrepancy between the global and voxel‐level analyses, further research should seek to replicate this finding.

This study is strengthened by the use of a longitudinal study design, allowing investigation of the temporal precedence of changes in morning–evening preference and symptoms of psychopathology. In addition, this is the first study to measure the effect of morning–evening preference on *changes* in brain development by utilizing multiple brain scans across adolescence. In contrast, our findings should be viewed in the context of several limitations. Direct measures of sleep, including presleep cognitions, were not collected in this study, precluding an opportunity to differentiate the respective roles of sleep and morning–evening preference on our selected outcomes. While previous studies have indicated that poor sleep is associated with deficits in brain structure (Jalbrzikowski et al., 2021; Kocevska et al., 2017; Takeuchi et al., 2018), literature in young adults highlights that neurobiological differences between morning‐ and evening‐oriented individuals exist even after controlling for the effect of sleep quality (Rosenberg et al., 2014). As such, it is difficult to disentangle the direct effect of these respective processes on neurobiological outcomes, and future research should seek to do so. Unfortunately, we were only able to estimate linear individual changes in morning–evening preference due to singularity restraints in the models. Future longitudinal studies with at least three time points should seek to model nonlinear random effects of sleep–wake behaviours across adolescence. Regarding the DWI acquisition, at *b* = 2,000, non‐Gaussian effects might affect FA estimation. Future studies could look at the Kurtosis model to observe separately the Gaussian effects in FA and non‐Gaussian in Kurtosis measures. In addition, our study assessed school students, limiting our ability to extrapolate to adolescents in vocational settings. Finally, we acknowledge that the presence of missing data and attrition may have limited the power of our analyses.

This study demonstrates that morning–evening preference changes nonlinearly across adolescence, and that intraindividual increases in eveningness are prospectively associated with changes in externalizing symptoms and trajectories of white matter development during this period. Findings from this study support relationships between morning–evening preference and developing neural circuitry, and indicate the temporal specificity of the relationship between increases in eveningness and externalizing psychopathology during adolescence. Given this evidence, public health intervention programmes might be suited to target evening‐oriented individuals early in development in order to prevent or ameliorate poorer mental and neurobiological health outcomes.

## Supporting information


**Appendix S1** Methods.
**Appendix S2**. Results.
**Table S1**. Descriptive statistics comparing individuals who were scanned at T3 compared to those who were not scanned at T3.
**Table S2**. Descriptive statistics comparing individuals who were scanned at T4 compared to those who were not scanned at T4.
**Table S3**. Descriptive statistics comparing individuals with parent‐reported externalizing symptom scores (as assessed by the CBCL) at T4 compared to those without parent‐reported externalizing symptom scores at T4.
**Table S4**. Model fit statistics for linear mixed models estimating the main effect of age on morning–evening preference across adolescence.
**Table S5**. Linear mixed‐effects model of the effect of age on morning–evening preference across adolescence (additionally controlling for pubertal status).
**Table S6**. Transitions between categories of morning–evening types across adolescence.
**Table S7**. Effect of change in morning–evening preference on parent‐reported externalizing symptoms at 19 years.
**Table S8**: Effect of change in morning–evening preference on anxiety symptoms at 19 years.
**Table S9**. Effect of change in morning–evening preference on depressive symptoms at 19 years.
**Table S10**: Effect of change in morning–evening preference on depressive symptoms at 19 years (additionally controlling for pubertal status).
**Table S11**. Effect of change in morning–evening preference on anxiety symptoms at 19 years (additionally controlling for pubertal status).
**Table S12**. Effect of change in morning–evening preference on externalizing symptoms at 19 years of age (additionally controlling for pubertal status).
**Table S13**. Effect of change in depression symptoms on morning–evening preference at 19 years of age.
**Table S14**. Effect of change in externalizing symptoms on morning–evening preference at 19 years of age.
**Table S15**. Effect of change in anxiety symptoms on morning–evening preference at 19 years of age.
**Table S16**. Effect of change in morning–evening preference on externalizing symptoms at 19 years of age (additionally controlling for recent substance use).
**Table S17**. Effect of change in morning–evening preference on self‐reported externalizing symptoms at 19 years.
**Table S18**. Effect of change in morning–evening preference on CBCL Delinquency subscale.
**Table S19**. Effect of change in morning–evening preference on CBCL Aggressive Behavior subscale.
**Table S20**. Effect of change in morning–evening preference on the annualized percent change (APC) in global fractional anisotropy (FA), from 17–19 years of age.
**Table S21**. Effect of change in morning–evening preference on the annualized percent change (APC) in global mean diffusivity (MD), from 17–19 years of age.
**Table S22**. Effect of annualized percentage change in global fractional anisotropy (FA) from 17–19 years of age on morning–evening preference at age 19 years.
**Figure S1**. Flow chart of participants and attrition of sample.
