# Supplementary material for: Development of morning–eveningness in adolescence: implications for brain development and psychopathology
Source: J Child Psychol Psychiatry. 2022 Nov 3;64(3):449–60. doi: 10.1111/jcpp.13718 (PMC10952670; doi:10.1111/jcpp.13718)
Supplement: Supplementary file 1 — Appendix S1 Methods. Appendix S2. Results. Table S1. Descriptive statistics comparing individuals who were scanned at T3 compared to those who were not scanned at T3. Table S2. Descriptive statistics comparing individuals who were scanned at T4 compared to those who were not scanned at T4. Table S3. Descriptive statistics comparing individuals with parent‐reported externalizing symptom scores (as assessed by the CBCL) at T4 compared to those without parent‐reported externalizing symptom scores at T4. Table S4. Model fit statistics for linear mixed models estimating the main effect of age on morning–evening preference across adolescence. Table S5. Linear mixed‐effects model of the effect of age on morning–evening preference across adolescence (additionally controlling for pubertal status). Table S6. Transitions between categories of morning–evening types across adolescence. Table S7. Effect of change in morning–evening preference on parent‐reported externalizing symptoms at 19 years. Table S8: Effect of change in morning–evening preference on anxiety symptoms at 19 years. Table S9. Effect of change in morning–evening preference on depressive symptoms at 19 years. Table S10: Effect of change in morning–evening preference on depressive symptoms at 19 years (additionally controlling for pubertal status). Table S11. Effect of change in morning–evening preference on anxiety symptoms at 19 years (additionally controlling for pubertal status). Table S12. Effect of change in morning–evening preference on externalizing symptoms at 19 years of age (additionally controlling for pubertal status). Table S13. Effect of change in depression symptoms on morning–evening preference at 19 years of age. Table S14. Effect of change in externalizing symptoms on morning–evening preference at 19 years of age. Table S15. Effect of change in anxiety symptoms on morning–evening preference at 19 years of age. Table S16. Effect of change in morning–evening preference on externalizing symptoms at [file JCPP-64-449-s001.docx]

**Supporting information**

**Appendix S1. Methods**

**Handling of missing data**

Participant data was obtained from a previous longitudinal cohort study (the Orygen Adolescent Development Study [OADS]). This study followed 245 individuals across four time points, with assessments at approximately 12, 15, 17 and 19 years of age. From this sample, 209 (101 male) individuals completed the reduced Morningness-Eveningness Questionnaire (rMEQ) 1-4 times, and were included as the final analytical sample. The number of individuals that completed the rMEQ varied at each wave, ranging from 58 (T2) to 152 (T1). Only a minority of individuals were asked to complete the rMEQ at T2, limiting the available data at this time-point substantially. To overcome this missingness, linear mixed models (LMMs) were selected to assess changes in rMEQ over time. LMMs provide an advantage over traditional repeated-measures analyses as they make use of all available data across time-points, even in the presence of missing data (Janmaat et al., 2019; Krueger & Tian, 2004). In addition, and to address missingness in other variables of interest, pairwise deletion was employed to maximise the number of data points in each analysis. The N included in each model is included in the table footnotes. A flow chart of participants and sample attrition is provided in Figure S1.

**Appendix S2. Results**

**Sensitivity Analysis**

A series of paired-sample t-tests revealed that the reduced sample that completed the rMEQ at T2 were significantly younger than those that did not complete the rMEQ at this time-point (t(207)=3.98, p<.001), but did not significantly differ on any other variables of interest (including sex, ancestry, BAI, CBCL and CESD scores). Further, there were no significant differences in estimated rMEQ slopes (indicating individual-level changes in rMEQ) between the groups (T1-T4 rMEQ slopes, Mann-Whitney U = 3858, p=0.324; T1-T3 rMEQ slopes, U=3609, p=0.481). This suggests that the reduction in sample size at T2 did not significantly bias or influence the estimation of age-associated changes in rMEQ. Further, given the reduction in sample size when including T2 pubertal status as a covariate, we also compared the estimated rMEQ slope for those with pubertal data to the larger cohort, and found no significant differences for either T1-T3 slopes or T1-T4 slopes (T1-T4 rMEQ slopes, Mann-Whitney U = 3284, p=0.119; T1-T3 rMEQ slopes, U=2727, p=0.115). These findings suggest that the analysis controlling for pubertal status was not significantly biased or influenced by individuals with pubertal data available.

Individuals that completed MRI scans and T3 and T4 were significantly younger than those that did not complete MRI scans at these waves (T3, t(207)=3.89, p<.001; T4, t(207)=3.61, p<.001); in addition, those scanned at T3 had significantly lower BAI (anxiety) scores than those not scanned (t(161)=2.15, p=0.033). Otherwise, individuals were comparable on variables of interest (see Tables S1 and S2). The difference in age is unlikely to significantly influence our findings, given we were assessing individual changes in FA only within the sample that were scanned. In addition, anxiety scores were not assessed in relation to FA. Lastly, we compared individuals with externalizing symptom scores at T4 with those who did not provide externalizing scores, given our limited N for this outcome variable (see Table S5). Individuals were comparable on all measures, except that individuals *without* externalizing scores were more evening-oriented (i.e. lower rMEQ scores; t(129)=-2.30, p=0.023). While this is a key outcome variable, the greater eveningness in the excluded sample would suggest that our results are attenuated somewhat, and that inclusion of the full sample might result in a stronger effect.

**Additional Mediation Models**

Mediation models were performed to examine whether white matter development mediated the relationship between rMEQ trajectory and psychopathology at 19 years of age (i.e. T4). Mediation models for predicting internalizing symptoms are reported here. For the model predicting T4 depression symptoms, neither path a (rMEQ trajectory on the mediator, APC in FA) nor path b (APC in FA on externalizing symptoms) were significant (path a, standardized B=0.19, t=1.75, p=0.083; *path b*, standardized B=0.03, t=0.22, p=0.825). The direct effect of rMEQ trajectory on depression (path c’) was not significant (standardized B=-0.09, t=-0.83, p=0.405), and the standardized indirect effect (a*b) was 0.19*0.03=0.006. The bootstrapped standardized indirect effect was 0.073 (SE 0.0048, 95% confidence interval -0.06-0.08). As the bootstrap confidence interval includes zero, the indirect effect is not significant, indicating white matter development did not mediate the relationship between trajectory of eveningness and depression symptoms at T4. For the model predicting T4 anxiety symptoms, path a (rMEQ trajectory on the mediator, APC in FA) was significant (standardized B=0.22, t=2.02, p=0.046), while path b (APC in FA on externalizing symptoms) was not significant (standardized B=0.07, t=0.63, p=0.532). The direct effect of rMEQ trajectory on anxiety (path c’) was not significant (standardized B=0.02, t=0.16, p=0.873). The standardized indirect effect (a*b) was 0.22*0.07=0.015. The bootstrapped standardized indirect effect was 0.015 (SE 0.03, 95% confidence interval -0.03-0.08). ). As the bootstrap confidence interval includes zero, the indirect effect is not significant, and thus white matter development did not mediate the relationship between trajectory of eveningness and anxiety at T4.

**Figure S1. Flow chart of participants and attrition of sample.**


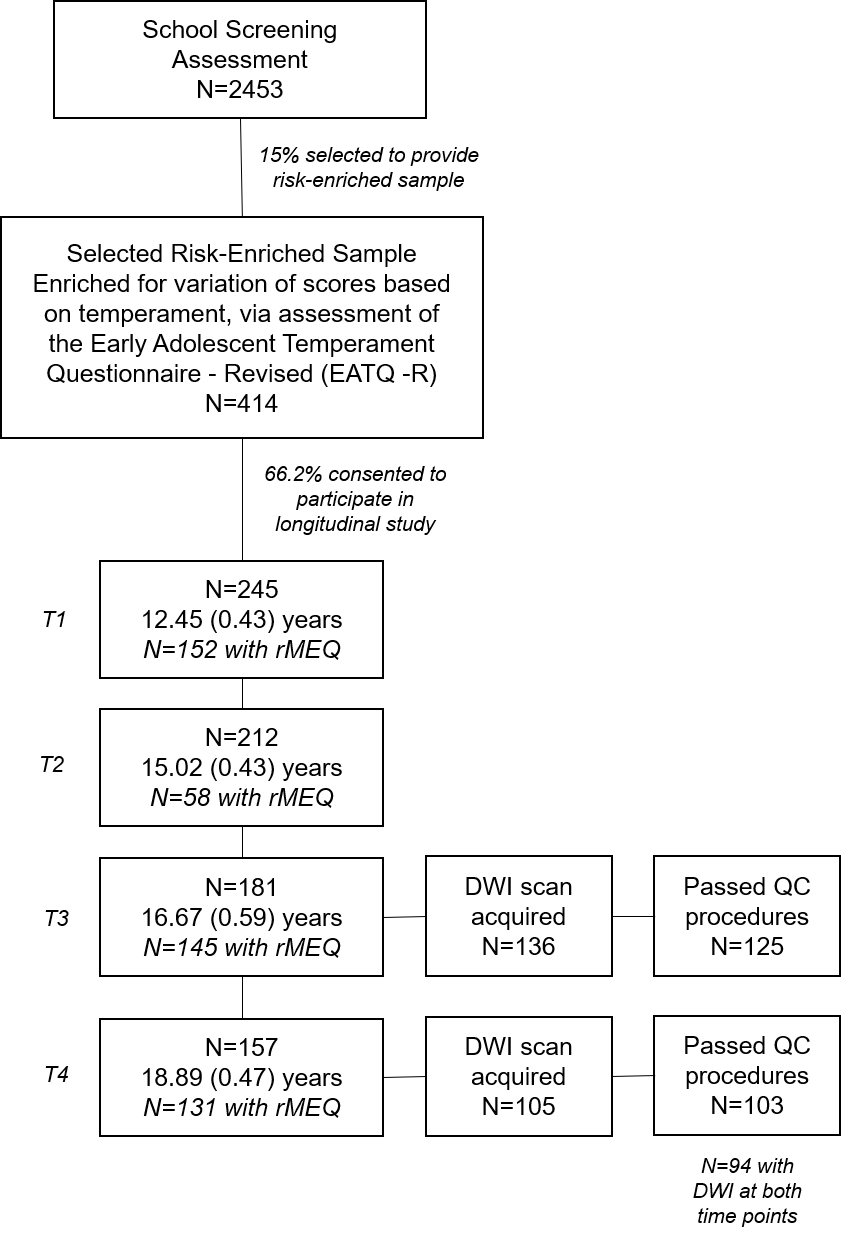


**Table S1. Descriptive statistics comparing individuals who were scanned at T3 compared to those who were not scanned at T3.**

|  | *Scanned at T3, N=94* | | *Not scanned at T3, N=115* | |  |
| --- | --- | --- | --- | --- | --- |
| Variable | Mean | SD | Mean | SD | *p* |
| Male, N (%) | 46 (49%) |  | 56 (49%) |  | 1 |
| Age | 16.5 | 0.53 | 16.84 | 0.63 | <0.001 |
| Ancestry*^a^* |  |  |  |  | 0.988 |
| Central European | 92% |  | 91% |  |  |
| Chinese | 8% |  | 9% |  |  |
| rMEQ | 13.49 | 1.68 | 13.3 | 1.72 | 0.514 |
| BAI | 7.79 | 7.14 | 10.73 | 10.24 | 0.033 |
| CBCL | 8.82 | 8.31 | 9.54 | 11.09 | 0.642 |
| CESD | 9.82 | 7.68 | 12.01 | 10.05 | 0.119 |

*^a^*Based on genetic ancestry markers. **All measures reflect T3 assessment. BAI,** Beck Anxiety Inventory; CBCL, Child Behavior Checklist, parent-report, externalizing subscale; CESD, Centre for Epidemiological Studies Depression Scale; rMEQ, reduced Morningness-Eveningness Questionnaire.

**Table S2. Descriptive statistics comparing individuals who were scanned at T4 compared to those who were not scanned at T4.**

|  | *Scanned at T4, N=94* | | *Not scanned at T4, N=115* | |  |
| --- | --- | --- | --- | --- | --- |
| Variable | Mean | SD | Mean | SD | *p* |
| Male, N (%) | 46 (49%) |  | 56 (49%) |  | 1 |
| Age | 18.8 | 0.47 | 19.07 | 0.42 | <0.001 |
| Ancestry*^a^* |  |  |  |  | 0.988 |
| Central European | 92% |  | 91% |  |  |
| Chinese | 8% |  | 9% |  |  |
| rMEQ | 13.28 | 1.74 | 13.55 | 1.61 | 0.376 |
| BAI | 7.28 | 8.27 | 6.77 | 8.1 | 0.694 |
| CBCL | 7.92 | 8.72 | 7.05 | 8.34 | 0.56 |
| CESD | 10.57 | 9.54 | 11.74 | 9.88 | 0.454 |

*^a^*Based on genetic ancestry markers. All **measures reflect T4 assessment. BAI,** Beck Anxiety Inventory; CBCL, Child Behavior Checklist, parent-report, externalizing subscale; CESD, Centre for Epidemiological Studies Depression Scale; rMEQ, reduced Morningness-Eveningness Questionnaire.

**Table S3. Descriptive statistics comparing individuals with parent-reported externalizing symptom scores (as assessed by the CBCL) at T4 compared to those without parent-reported externalizing symptom scores at T4.**

|  | *Completed CBCL at T4, N=136* | | *Did not complete CBCL at T4, N=73* | |  |
| --- | --- | --- | --- | --- | --- |
| Variable | Mean | SD | Mean | SD | *p* |
| Male, N (%) | 67 (49%) |  | 35 (48%) |  | 0.971 |
| Age | 18.9 | 0.49 | 18.96 | 0.36 | 0.505 |
| Ancestry*^a^* |  |  |  |  | 0.163 |
| Central European | 94% |  | 87% |  |  |
| Chinese | 6% |  | 13% |  |  |
| BAI | 7.13 | 8.47 | 6.77 | 6.87 | 0.827 |
| rMEQ | 13.53 | 1.57 | 12.58 | 2.14 | 0.023 |
| CESD | 10.76 | 9.66 | 12.45 | 9.81 | 0.398 |
| Substance use frequency | 40.21 | 43.12 | 58.29 | 50.56 | 0.054 |
| Youth Self Report^b^ | 11.6 | 7.51 | 12.12 | 6.88 | 0.751 |

*^a^*Based on genetic ancestry markers. *^b^*Reflects self-reported externalizing symptoms. All **measures reflect T4 assessment. BAI,** Beck Anxiety Inventory; CBCL, Child Behavior Checklist, externalizing subscale; CESD, Centre for Epidemiological Studies Depression Scale; rMEQ, reduced Morningness-Eveningness Questionnaire.

**Table S4. Model fit statistics for linear mixed models estimating the main effect of age on morning-evening preference across adolescence.**

| ***Model*** | ***Fixed effect of age*** | ***AIC*** | ***BIC*** | ***Model comparison*** | ***L. Ratio*** | ***p*** |
| --- | --- | --- | --- | --- | --- | --- |
| **1** | **Linear** | **1775.13** | **1791.69** |  |  |  |
| **2** | **Quadratic** | **1770.10** | **1794.94** | **1 vs 2** | **9.030** | **0.0109** |
| **3** | **Cubic** | **1772.10** | **1801.08** | **2 vs 3** | **0.004** | **0.9475** |

**Models compared via analysis of variance (ANOVA). Sex included as covariate. AIC, Akaike Information Criterion; BIC, Bayesian Information Criterion; L-ratio, Likelihood ratio.**

**Table S5: Linear mixed-effects model of the effect of age on morning-evening preference across adolescence (additionally controlling for pubertal status).**

| *Predictors* | *Estimates* | *Std. Error* | *Standardized B* | *t* | *p* |
| --- | --- | --- | --- | --- | --- |
| Intercept | 26.85 | 3.59 | 0.01 | 7.48 | **<.001** |
| Age | -1.50 | 0.46 | -2.15 | -3.24 | **0.001** |
| Age^2^ | 0.04 | 0.01 | 1.87 | 2.82 | **0.005** |
| Sex | 0.02 | 0.21 | 0.01 | 0.11 | **0.909** |
| Pubertal status (T2) | -0.01 | 0.16 | -0.00 | -0.03 | 0.975 |
| Marginal R^2^ / Conditional R^2^ | | | 0.094 / 0.345 | |  |

**Linear mixed effects modelled using the *nlme* package in R.** Sex and pubertal status included as covariates. N observations = 383; N individuals = 152.

**Table S6. Transitions between categories of morning-evening types across adolescence.**

| *Time point* | *T1* | *T2* | *T3* | *T4* |
| --- | --- | --- | --- | --- |
| *Available data (N)* | *150* | *57* | *147* | *131* |
| M-type | 5 (3%) | 0 | 0 | 0 |
| I-type | 143 (95%) | 54 (95%) | 132 (90%) | 115 (88%) |
| E-type | 2 (1%) | 3 (5%) | 15 (10%) | 16 (12%) |
| *Transition in subsequent time-point (N)* | | | | |
| M to I | 2 | - | - | - |
| M to E | 0 | - | - | - |
| M to M | 0 | - | - | - |
| I to E | 3 | 3 | 11 | - |
| I to M | 0 | 0 | 0 | - |
| I to I | 34 | 38 | 82 | - |
| E to M | 0 | 0 | 0 | - |
| E to I | 0 | 2 | 7 | - |
| E to E | 0 | 0 | 2 | - |

**E, evening-type; I, intermediate-type; M, morning-type. Note: due to missing data, not all individuals transition to subsequent time-points.**

**Table S7. Effect of change in morning-evening preference on parent-reported externalizing symptoms at 19 years.**

| *Predictors* | *Estimates* | *Std. Error* | *Standardized B* | *t* | *p* |
| --- | --- | --- | --- | --- | --- |
| Intercept | 2.38 | 0.70 | -0.08 | 3.41 | 0.001 |
| Change in rMEQ (T1-T4) | -10.85 | 3.27 | -0.16 | -3.32 | **0.001** |
| Sex (Male) | -1.27 | 0.80 | -0.16 | -1.59 | 0.115 |
| Baseline CBCL | 0.47 | 0.05 | 0.44 | 8.92 | <.001 |
| F-statistic | 29.76 |  |  |  | **<.001** |
| Weighted R^2^*^a^* | 0.611 |  |  |  |  |

*^a^*Weighted R^2^ calculated due to use of weighted Huber Regressions in analysis**.** CBCL, Child Behavior Checklist, parent-report, externalizing scale; rMEQ, reduced Morningness-Eveningness Questionnaire. Sex included as covariate. Values in bold survive Bonferroni correction, α set at 0.05/6 = 0.0083. N observations = 114.

**Table S8: Effect of change in morning-evening preference on anxiety symptoms at 19 years.**

| *Predictors* | *Estimates* | *Std. Error* | *Standardized B* | | | *t* | *p* |
| --- | --- | --- | --- | --- | --- | --- | --- |
| Intercept | 4.62 | 0.72 | -0.09 | | | 6.40 | **<.001** |
| Change in rMEQ (T1-T4) | -1.64 | 3.40 | -0.03 | | | -0.48 | 0.632 |
| Sex (Male) | -2.19 | 0.88 | -0.26 | | | -2.48 | **0.014** |
| Baseline BAI | 0.22 | 0.06 | 0.21 | | | 3.94 | **<.001** |
| F-statistic | 6.04 |  |  |  |  |  | **<.001** |
| Weighted R^2^*^a^* | 0.305 |  |  |  |  |  | |

*^a^*Weighted R^2^ calculated due to use of weighted Huber Regressions in analysis**. BAI,** Beck Anxiety Inventory; rMEQ, reduced Morningness-Eveningness Questionnaire. Sex included as covariate. N observations = 149.

**Table S9: Effect of change in morning-evening preference on depressive symptoms at 19 years.**

| *Predictors* | *Estimates* | *Std. Error* | *Standardized B* | | | *t* | *p* |
| --- | --- | --- | --- | --- | --- | --- | --- |
| Intercept | 6.69 | 1.19 | -0.03 | | | 5.62 | **<.001** |
| Change in rMEQ (T1-T4) | -2.60 | 5.22 | -0.04 | | | -0.50 | 0.619 |
| Sex (Male) | -2.36 | 1.36 | -0.25 | | | -1.74 | 0.085 |
| Baseline CESD | 0.37 | 0.08 | 0.34 | | | 4.82 | **<.001** |
| F-statistic | 8.34 |  |  |  |  |  | **<.001** |
| Weighted R^2^*^a^* | 0.285 |  |  |  |  |  | |

*^a^*Weighted R^2^ calculated due to use of weighted Huber Regressions in analysis**.** CESD, Centre for Epidemiological Studies Depression Scale; rMEQ, reduced Morningness-Eveningness Questionnaire. Sex included as covariate. N observations = 127.

**Table S10: Effect of change in morning-evening preference on depressive symptoms at 19 years (additionally controlling for pubertal status).**

| *Predictors* | *Estimates* | *Std. Error* | *Standardized B* | *t* | *p* |
| --- | --- | --- | --- | --- | --- |
| Intercept | 5.58 | 5.00 | -0.04 | 1.11 | 0.268 |
| Change in rMEQ (T1-T4) | -4.59 | 6.12 | -0.06 | -0.75 | 0.455 |
| Pubertal status (T2) | 0.21 | 1.17 | 0.01 | 0.18 | 0.859 |
| Sex (Male) | -2.45 | 1.53 | -0.25 | -1.60 | 0.113 |
| Baseline CESD | 0.36 | 0.08 | 0.33 | 4.28 | <.001 |
| F-statistic | 5.345 |  |  |  | <.001 |
| Weighted R^2^*^a^* | 0.320 |  |  |  | |

*^a^*Weighted R^2^ calculated due to use of weighted Huber Regressions in analysis**.** CESD, Centre for Epidemiological Studies Depression Scale; rMEQ, reduced Morningness-Eveningness Questionnaire. Sex and pubertal status included as covariates. N observations = 97.

**Table S11: Effect of change in morning-evening preference on anxiety symptoms at 19 years (additionally controlling for pubertal status).**

| *Predictors* | *Estimates* | *Std. Error* | *Standardized B* | *t* | *p* |
| --- | --- | --- | --- | --- | --- |
| Intercept | 1.49 | 3.08 | -0.06 | 0.48 | 0.630 |
| Change in rMEQ (T1-T4) | -3.90 | 4.01 | -0.06 | -0.97 | 0.333 |
| Pubertal status (T2) | 0.96 | 0.73 | 0.08 | 1.31 | 0.193 |
| Sex (Male) | -2.59 | 1.01 | -0.31 | -2.57 | 0.011 |
| Baseline BAI | 0.14 | 0.06 | 0.13 | 2.24 | 0.027 |
| F-statistic | 3.00 |  |  |  | **0.021** |
| Weighted R^2^*^a^* | 0.298 |  |  |  |  |

*^a^*Weighted R^2^ calculated due to use of weighted Huber Regressions in analysis**. BAI,** Beck Anxiety Inventory; rMEQ, reduced Morningness-Eveningness Questionnaire. Sex and pubertal status included as covariates. N observations = 115.

**Table S12. Effect of change in morning-evening preference on externalizing symptoms at 19 years of age (additionally controlling for pubertal status).**

| *Predictors* | *Estimates* | *Std. Error* | *Standardized B* | *t* | *p* |
| --- | --- | --- | --- | --- | --- |
| Intercept | -0.80 | 2.88 | -0.01 | -0.28 | 0.782 |
| Change in rMEQ (T1-T4) | -14.76 | 3.44 | -0.25 | -4.29 | **<.001** |
| Pubertal status (T2) | 1.01 | 0.66 | 0.08 | 1.52 | 0.133 |
| Sex (Male) | -1.91 | 0.88 | -0.25 | -2.16 | 0.033 |
| Baseline CBCL | 0.42 | 0.06 | 0.41 | 7.23 | <.001 |
| F-statistic | 19.91 |  |  |  | <.001 |
| Weighted R^2^*^a^* | 0.613 |  |  |  |  |

*^a^*Weighted R^2^ calculated due to use of weighted Huber Regressions in analysis**.** CBCL, Child Behavior Checklist, parent-report, externalizing subscale; rMEQ, reduced Morningness-Eveningness Questionnaire. Sex and pubertal status included as covariates. Values in bold survive Bonferroni correction, α set at 0.05/6 = 0.0083. N observations = 93.

**Table S13. Effect of change in depression symptoms on morning-evening preference at 19 years of age.**

| *Predictors* | *Estimates* | *Std. Error* | *Standardized B* | *t* | *p* |
| --- | --- | --- | --- | --- | --- |
| Intercept | 9.75 | 1.53 | 0.12 | 6.38 | **<.001** |
| Change in CESD (T1-T4) | 0.17 | 0.12 | 0.14 | 1.46 | 0.148 |
| Sex (Male) | -0.03 | 0.33 | -0.02 | -0.08 | 0.934 |
| Baseline rMEQ | 0.25 | 0.10 | 0.23 | 2.46 | **0.016** |
| F-statistic | 2.62 |  |  |  | **0.06** |
| Weighted R^2^*^a^* | 0.164 |  |  |  |  |

*^a^*Weighted R^2^ calculated due to use of weighted Huber Regressions in analysis**.** CESD, Centre for Epidemiological Studies Depression Scale; rMEQ, reduced Morningness-Eveningness Questionnaire. Sex included as covariate. N observations = 86.

**Table S14. Effect of change in externalizing symptoms on morning-evening preference at 19 years of age.**

| *Predictors* | *Estimates* | *Std. Error* | *Standardized B* | *t* | *p* |
| --- | --- | --- | --- | --- | --- |
| Intercept | 9.76 | 1.49 | 0.14 | 6.56 | <.001 |
| Change in CBCL (T1-T4) | -0.12 | 0.45 | -0.03 | -0.27 | 0.789 |
| Sex (Male) | -0.16 | 0.33 | -0.09 | -0.48 | 0.635 |
| Baseline rMEQ | 0.26 | 0.10 | 0.24 | 2.57 | 0.012 |
| F-statistic | 2.22 |  |  |  | **0.09** |
| Weighted R^2^*^a^* | 0.122 |  |  |  |  |

*^a^*Weighted R^2^ calculated due to use of weighted Huber Regressions in analysis**.** CBCL, Child Behavior Checklist, parent-report, externalizing subscale; rMEQ, reduced Morningness-Eveningness Questionnaire. Sex included as covariate. N observations = 81.

**Table S15. Effect of change in anxiety symptoms on morning-evening preference at 19 years of age.**

| *Predictors* | *Estimates* | *Std. Error* | *Standardized B* | *t* | *p* |
| --- | --- | --- | --- | --- | --- |
| Intercept | 10.05 | 1.49 | 0.11 | 6.75 | **<.001** |
| Change in BAI (T1-T4) | 0.57 | 0.63 | 0.08 | 0.91 | 0.367 |
| Sex (Male) | -0.04 | 0.32 | -0.02 | -0.12 | 0.906 |
| Baseline rMEQ | 0.25 | 0.10 | 0.22 | 2.45 | **0.017** |
| F-statistic | 2.18 |  |  |  | **0.10** |
| Weighted R^2^*^a^* | 0.129 |  |  |  |  |

*^a^*Weighted R^2^ calculated due to use of weighted Huber Regressions in analysis**. BAI,** Beck Anxiety Inventory; rMEQ, reduced Morningness-Eveningness Questionnaire. Sex included as covariate. N observations = 139.

**Table S16. Effect of change in morning-evening preference on externalizing symptoms at 19 years of age (additionally controlling for recent substance use).**

| *Predictors* | *Estimates* | *Std. Error* | *Standardized B* | *t* | *p* |
| --- | --- | --- | --- | --- | --- |
| Intercept | 1.23 | 0.74 | -0.07 | 1.67 | 0.098 |
| Change in rMEQ (T1-T4) | -9.10 | 3.13 | -0.16 | -2.91 | **0.004** |
| Sex (Male) | -0.63 | 0.77 | -0.09 | -0.82 | 0.413 |
| Baseline CBCL | 0.41 | 0.05 | 0.41 | 7.74 | <.001 |
| Substance use frequency | 0.04 | 0.01 | 0.23 | 4.28 | <.001 |
| F-statistic | 26.52 |  |  |  | <.001 |
| Weighted R^2^*^a^* | 0.590 |  |  |  |  |

*^a^*Weighted R^2^ calculated due to use of weighted Huber Regressions in analysis**.**  Substance use frequency defined as number of days the individual consumed cigarettes, alcohol or cannabis in the past month. CBCL, Child Behavior Checklist, parent-report, externalizing scale; rMEQ, reduced Morningness-Eveningness Questionnaire. Values in bold survive Bonferroni correction, α set at 0.05/6 = 0.0083. N observations = 104.

**Table S17.** **Effect of change in morning-evening preference on self-reported externalizing symptoms at 19 years.**

| *Predictors* | *Estimates* | *Std. Error* | *Standardized B* | *t* | *p* |
| --- | --- | --- | --- | --- | --- |
| Intercept | 8.74 | 1.04 | -0.01 | 8.39 | <.001 |
| Change in rMEQ (T1-T4) | -10.57 | 4.88 | -0.18 | -2.17 | 0.032 |
| Sex (Male) | -0.56 | 1.24 | -0.07 | -0.45 | 0.655 |
| Baseline CBCL*^a^* | 0.35 | 0.09 | 0.34 | 4.14 | <.001 |
| F-statistic | 8.78 |  |  |  | <.001 |
| Weighted R^2^*^a^* | 0.214 |  |  |  |  |

*^a^*Model covaries for parent-reported CBCL as self-reported externalizing symptoms were not assessed at baseline. *^b^*Weighted R^2^ calculated due to use of weighted Huber Regressions in analysis. CBCL, Child Behavior Checklist, parent-report, externalizing scale; rMEQ, reduced Morningness-Eveningness Questionnaire. Sex included as covariate. N observations = 127.

**Table S18. Effect of change in morning-evening preference on CBCL Delinquency subscale.**

| *Predictors* | *Estimates* | *Std. Error* | *Standardized B* | *t* | *p* |
| --- | --- | --- | --- | --- | --- |
| Intercept | 0.72 | 0.29 | -0.13 | 2.48 | **0.015** |
| Change in rMEQ (T1-T4) | -3.14 | 1.27 | -0.12 | -2.47 | **0.015** |
| Sex (Male) | -0.27 | 0.32 | -0.08 | -0.84 | 0.404 |
| Baseline CBCL Total | 0.09 | 0.01 | 0.31 | 6.51 | **<.001** |
| F-statistic | 16.53 |  |  |  | **<.001** |
| Weighted R^2^*^a^* | 0.428 |  |  |  |  |

*^a^*Weighted R^2^ calculated due to use of weighted Huber Regressions in analysis**.** CBCL, Child Behavior Checklist, parent-report, externalizing scale; rMEQ, reduced Morningness-Eveningness Questionnaire. Sex included as covariate. N observations = 122.

Table S19. Effect of change in morning-evening preference on CBCL Aggressive Behavior subscale.

| *Predictors* | *Estimates* | *Std. Error* | *Standardized B* | *t* | *p* |
| --- | --- | --- | --- | --- | --- |
| Intercept | 1.74 | 0.55 | -0.05 | 3.15 | **0.002** |
| Change in rMEQ (T1-T4) | -6.52 | 2.43 | -0.15 | -2.69 | **0.008** |
| Sex (Male) | -1.07 | 0.61 | -0.19 | -1.77 | 0.080 |
| Baseline CBCL Total | 0.21 | 0.03 | 0.41 | 7.59 | **<.001** |
| F-statistic | 21.95 |  |  |  | **<.001** |
| Weighted R^2^*^a^* | 0.514 |  |  |  |  |

*^a^*Weighted R^2^ calculated due to use of weighted Huber Regressions in analysis**.** CBCL, Child Behavior Checklist, parent-report, externalizing scale; rMEQ, reduced Morningness-Eveningness Questionnaire. Sex included as covariate. N observations = 122.

**Table S20. Effect of c**hange in morning-evening preference on the annualized percent change (APC) in global fractional anisotropy (FA), from 17-19 years of age.

| *Predictors* | *Estimates* | *Std. Error* | *Standardized B* | *t* | *p* |
| --- | --- | --- | --- | --- | --- |
| Intercept | 0.21 | 0.09 | -0.11 | 2.37 | 0.020 |
| Change in rMEQ (T1-T3) | 22.87 | 9.67 | 0.24 | 2.36 | 0.020 |
| Sex (Male) | 0.08 | 0.13 | 0.12 | 0.60 | 0.549 |
| F-statistic | 2.62 |  |  |  | 0.057 |
| Weighted R^2^*^a^* | 0.164 |  |  |  |  |

*^a^*Weighted R^2^ calculated due to use of weighted Huber Regressions in analysis**. rMEQ,** reduced Morningness-Eveningness Questionnaire**.** Sex included as covariate. N observations = 88.

**Table S21. Effect of c**hange in morning-evening preference on the annualized percent change (APC) in global mean diffusivity (MD), from 17-19 years of age.

| *Predictors* | *Estimates* | *Std. Error* | *Standardized B* | *t* | *p* |
| --- | --- | --- | --- | --- | --- |
| (Intercept) | -0.42 | 0.10 | 0.13 | -4.26 | <.001 |
| Change in rMEQ (T1-T3) | 9.16 | 10.85 | 0.07 | 0.84 | 0.401 |
| Sex (Male) | -0.07 | 0.14 | -0.08 | -0.48 | 0.632 |
| F-statistic | 0.555 |  |  |  | 0.576 |
| Weighted R^2^*^a^* | 0.058 |  |  |  |  |

*^a^*Weighted R^2^ calculated due to use of weighted Huber Regressions in analysis**. rMEQ,** reduced Morningness-Eveningness Questionnaire**.** Sex included as covariate. N observations = 88.

**Table S22. Effect of annualized percentage change in global fractional anisotropy (FA) from 17-19 years of age on morning-evening preference at age 19 years.**

| *Predictors* | *Estimates* | *Std. Error* | *Standardized B* | *t* | *p* |
| --- | --- | --- | --- | --- | --- |
| (Intercept) | 9.00 | 1.80 | 0.08 | 4.99 | <.001 |
| APC in FA (17-19yrs) | 0.15 | 0.34 | 0.05 | 0.42 | 0.674 |
| Sex (Male) | -0.13 | 0.41 | -0.07 | -0.32 | 0.751 |
| rMEQ at 17 years*^a^* | 0.32 | 0.13 | 0.29 | 2.43 | 0.018 |
| F-statistic | 2.356 |  |  |  | 0.080 |
| Weighted R^2^*^b^* | 0.122 |  |  |  |  |

*^a^*rMEQ at 17 years included as baseline measure. *^b^*Weighted R^2^ calculated due to use of weighted Huber Regressions in analysis. **rMEQ,** reduced Morningness-Eveningness Questionnaire**.** Sex included as covariate. N observations = 67.

**References**

Janmaat, C. J., Van Diepen, M., Tsonaka, R., Jager, K. J., Zoccali, C., & Dekker, F. W. (2019). Pitfalls of linear regression for estimating slopes over time and how to avoid them by using linear mixed-effects models. *Nephrology Dialysis Transplantation*, *34*(4), 56–566. https://doi.org/10.1093/ndt/gfy128

Krueger, C., & Tian, L. (2004). A comparison of the general linear mixed model and repeated measures ANOVA using a dataset with multiple missing data points. *Biological Research for Nursing*, *6*(2), 151–157. https://doi.org/10.1177/1099800404267682
